# Audit to assess discussion of sexual dysfunction for new patients entering a community mental health recovery service

**DOI:** 10.1192/bjo.2021.210

**Published:** 2021-06-18

**Authors:** Yasmin Abbasi, Dina Robertson, Amarachi Anosike, Margaret Pearson, Kevin Pankhurst, Jane Perera

**Affiliations:** 1 Surrey and borders partnership nhs trust; 2 Medical student placement at surrey and borders partnership nhs trust

## Abstract

**Aims:**

Sexual dysfunction should be enquired about as a symptom of mental health disorders and as side effects of commonly used psychotropic drugs. We audited against NICE guidelines the record of sexual dysfunction discussion at initial assessment and follow-up by the community mental health recovery service (CMHRS).

**Background:**

Research reports that sexual dysfunction occurs more often in individuals with serious mental illnesses including depression and schizophrenia. Sexual dysfunction is also a reported side effect of antidepressant and antipsychotic medications. NICE guidelines recommend assessment of biological symptoms of mental health disorders and discussion of potential side effects of treatments being considered prior to initiation and at follow-up.

**Method:**

Our sample consisted of 71 patients, all new patient assessments from referrals made to CMHRS between January 1st and March 31st 2019.

We reviewed all initial assessment and follow-up electronic notes and any correspondence generated from these meetings.

**Result:**

Our results showed that no record was made of sexual dysfunction as present or absent by health care professionals (HCPs) completing initial assessment or follow-up.

We surveyed the HCPs from the team and observed a high level of confidence in discussing sexual dysfunction and high self report of this discussion being conducted.

**Conclusion:**

Our audit results show no records of the discussion of sexual dysfunction, we held to the principal that in absence of record the discussion did not take place. Our survey results suggested that HCPs were confident they do assess for sexual dysfunction. We wondered, therefore, if HCPs would be less likely to make record in the event that symptoms are denied, recognizing that the list of potential symptoms and side effects is extensive and documentation of all negative results would be time consuming.

Our audit results may show then, that sexual dysfunction is not present in any of the sample; however this would contrast to research findings of higher than average rates of sexual dysfunction in groups with serious mental illness and those using antidepressants or antipsychotics.

We propose further assessment is needed for the disparity between our and recognised rates of sexual dysfunction.

We propose the standard that recording ‘absence of biological symptoms’ of mental health disorders or recorded supply of medicine information leaflets are adequate record. We also made suggestions for training and recording to assist HCPs initial assessment.

